# Remote outcome measures in Alzheimer's disease clinical trials: A call to action

**DOI:** 10.1016/j.tjpad.2026.100528

**Published:** 2026-02-27

**Authors:** Gustavo A. Jimenez-Maggiora

**Affiliations:** Epstein Family Alzheimer’s Therapeutic Research Institute, University of Southern California, San Diego, CA, United States

The introduction of disease‑modifying therapies has shifted the landscape of Alzheimer’s disease research, yet clinical trials still rely heavily on frequent in‑person assessments. This model has become difficult to sustain and may limit participation by reducing representation, increasing burden, and excluding individuals who might otherwise enroll. The COVID‑19 pandemic underscored these limitations and demonstrated that remote assessments can support participant retention and data quality. Concurrent advances in artificial intelligence and digital health indicate the feasibility of remote tools that could augment future research practices. Developing and validating remote outcome measures is, therefore, a scientific, ethical, and operational priority that may help reduce disparities, improve the efficiency of therapeutic development, and expand accessibility for patients.

These challenges are reflected in the practical demands of Alzheimer’s disease clinical trials, which require considerable time and effort from participants and care partners. Many families attend multiple in‑person visits over periods approaching two years, and in some cases extending beyond four years, while managing extensive testing, travel requirements, and disruptions to daily routines. As this burden has increased, the limitations of the traditional in‑person model have become more evident, suggesting the potential value of approaches that maintain scientific rigor while reducing participant burden.

Periodic clinic visits provide brief observations of a condition that changes over time, which may reduce sensitivity to early treatment effects and may limit the ability to characterize trajectories of cognitive decline. This approach may also affect representativeness, as rural residents, working‑age adults, and individuals with limited resources may be less likely to participate, resulting in samples that may not reflect populations most affected by Alzheimer’s disease. The logistical demands of repeated travel, time away from work, and caregiving coordination can contribute to dropout and complicate recruitment. In addition, staffing requirements and facility costs can increase trial expenditures and limit the number of therapies that can be assessed.

Remote outcome measures offer an approach for collecting cognitive, functional, behavioral, and physiological data through smartphones, tablets, wearables, and other connected devices [1]. These tools extend beyond digital versions of clinic‑based assessments. Smartphone‑based cognitive tasks can measure multiple domains with fine temporal resolution, and speech and language samples analyzed with natural language processing can detect early changes that may be missed by conventional testing [2,3]. Passive sensing from accelerometers, GPS data, and device‑use patterns can provide information about mobility, navigation, social engagement, and daily activities [4]. Actigraphy and related monitoring methods can identify early indicators of agitation, sleep disruption, apathy, depression, and other neuropsychiatric symptoms. Wearable devices support continuous physiologic and safety monitoring through measures such as heart rate, heart rate variability, sleep parameters, and potentially blood pressure, with fall‑detection capabilities and health‑system integration enabling timely follow‑up when needed. Together, these approaches allow for high‑frequency, ecologically grounded measurement; may reduce participant burden; broaden access beyond geographic limitations; support ongoing safety monitoring; lower per‑assessment costs; and help maintain retention by reducing logistical demands.

Several factors indicate that this is an opportune time to revise trial practices. Regulatory agencies now recognize digital endpoints and provide guidance for remote data collection, with parallel frameworks emerging internationally. Technological readiness is high, with widespread use of smartphones and wearables, clinically capable sensors, and established cloud infrastructure. Clinically, the availability of disease‑modifying therapies increases the need for earlier detection and more sensitive outcome measures, and remote digital biomarkers may help identify preclinical changes sooner than conventional assessments [5,6]. Equity considerations also support modernization, as remote approaches may reduce travel‑related barriers for underrepresented groups. Economic pressures reinforce this shift, given the high cost of Phase III trials and the substantial contribution of in‑person assessments to overall expenditures. Increased industry activity and academic innovation are accelerating development.

Several challenges must be addressed to support the broad adoption of remote outcome measures. Validation remains essential: traditional assessments are supported by decades of empirical research, whereas digital measures have a comparatively limited evidence base. Accelerated but rigorous studies are therefore needed to establish reliability, validity, responsiveness, and interpretability. Standardization is also required. The current landscape is fragmented across devices, platforms, data standards, and analytic methods. Developing consensus on core measures, platform specifications, quality‑control procedures, and shared analytic frameworks would improve comparability across studies and facilitate regulatory evaluation.

Ensuring technology access and digital literacy will be important so that technological barriers do not replace geographic ones. Some participants may require provisioned devices, training, and ongoing support. Privacy and security also require sustained attention, as continuous monitoring produces sensitive data; strong safeguards and clear consent processes are needed to maintain trust. High‑frequency, multimodal data introduce analytic complexity, including missingness and device‑related variability, underscoring the need for advanced modeling and standardized guidelines. Digital measures may also demonstrate clinical meaningfulness. Finally, concordance studies will be needed to clarify how digital metrics relate to traditional endpoints and how they may complement or eventually supplant them.

A coordinated multi‑stakeholder effort is needed to support the adoption of remote outcome measures. This includes conducting multi‑site validation studies; establishing shared standards for digital assessments, devices, data, and analytics; maintaining regulatory engagement to refine evidentiary expectations; advancing equity through implementation research; strengthening data infrastructure and analytic capacity; and using pilot studies to evaluate feasibility and operational workflows.

We can envision a future prevention trial that takes advantage of remote outcome measures. In such a trial, participants nationwide, including those in rural areas, complete screening entirely at home. Screening is enriched by combining a fifteen‑minute smartphone‑based cognitive assessment with a plasma p‑tau217 test obtained locally, followed by a telehealth visit to confirm eligibility [5,6]. In‑person safety visits and MRIs are retained, but informative endpoints are collected remotely through weekly smartphone‑based cognitive tasks, continuous smartwatch monitoring, monthly partner‑reported functional assessments, and weekly speech samples analyzed with natural language processing[2,3]. An agentic voice‑based system, accessible via smartphone or smart speaker, conducts structured conversational assessments that estimate Alzheimer’s severity and progression, offering a scalable, low‑burden complement to other digital measures. A validated in-clinic composite digital cognitive score might serve as the primary endpoint while secondary endpoints include digital measures of function, sleep efficiency, and activity patterns [4]. Automated alerts prompt safety follow‑up when concerning decline is detected, and participants demonstrating stable or improving performance and consistent intervention compliance receive supportive engagement feedback. In this model, recruitment is completed faster, dropout is reduced, representativeness increases, per‑participant costs decline, and treatment effects are detected sooner, enabling earlier regulatory submission. All required components already exist or are in advanced development, as evidenced by the decentralized TRAILBLAZER ALZ-3 trial in preclinical AD [7]; coordinated integration is the next step.

The development, validation, and implementation of remote outcome measures will require coordinated efforts from regulators, funders, industry partners, academic institutions, technology developers, patient groups, and professional societies. Remote digital measures are likely to enable trials that are more efficient, more inclusive, and more reflective of real‑world patient experience.

## Declaration of the use of generative AI

During the preparation of this work, the author used Microsoft Copilot (ChatGPT‑5.2) and Grammarly solely for language editing to enhance clarity and readability. After using these tools, the author reviewed and revised the text as needed and takes full responsibility for the content of the publication.

## CRediT authorship contribution statement

**Gustavo A. Jimenez-Maggiora:** Writing – original draft.

## Declaration of competing interest

The authors declare the following financial interests/personal relationships which may be considered as potential competing interests:

Gustavo A. Jimenez-Maggiora reports a relationship with National Institute on Aging that includes: funding grants. Gustavo A. Jimenez-Maggiora reports a relationship with Alzheimer’s Association that includes: funding grants. Gustavo A. Jimenez-Maggiora reports a relationship with American Heart Association Inc that includes: funding grants. Gustavo A. Jimenez-Maggiora reports a relationship with Eli Lilly and Company that includes: funding grants. Gustavo A. Jimenez-Maggiora reports a relationship with Eisai Co Ltd that includes: funding grants. Author has received funding or support from Gates Ventures - GJM; Author has served as a reviewer for this journal - GJM If there are other authors, they declare that they have no known competing financial interests or personal relationships that could have appeared to influence the work reported in this paper.
